# Profiling and metaanalysis of epidermal keratinocytes responses to epidermal growth factor

**DOI:** 10.1186/1471-2164-14-85

**Published:** 2013-02-08

**Authors:** Miroslav Blumenberg

**Affiliations:** 1 The R. O. Perelman Department of Dermatology, Department of Biochemistry and Molecular Pharmacology, and the NYU Cancer Institute, NYU Langone Medical Center, 10016, New York, NY, USA

**Keywords:** Apoptosis, Cornification, Differentiation, Inflammation, Microarrays, Motility, Proliferation

## Abstract

**Background:**

One challenge of systems biology is the integration of new data into the preexisting, and then re-interpretation of the integrated data. Here we use readily available metaanalysis computational methods to integrate new data on the transcriptomic effects of EGF in primary human epidermal keratinocytes with preexisting transcriptomics data in keratinocytes and in EGF-treated non-epidermal cell types.

**Results:**

We find that EGF promotes keratinocyte proliferation, attachment and motility and, surprisingly, induces DUSPs that attenuate the EGF signal. Our metaanalysis identified overlapping effects of EGF with those of IL-1 and IFNγ, activators of keratinocyte in inflammation and wound healing. We also identified the genes and pathways suppressed by EGF but induced by agents promoting epidermal differentiation. Metaanalysis comparison with the EGF effects in other cell types identified extensive similarities between responses in keratinocytes and in other epithelial cell types, but specific differences with the EGF effects in endothelial cells, and in transformed, oncogenic epithelial cell lines.

**Conclusions:**

This work defines the specific transcriptional effects of EGF on human epidermal keratinocytes. Our approach can serve as a suitable paradigm for integration of new omics data into preexisting databases and re-analysis of the integrated data sets.

## Background

Systems biology aims to integrate different levels and types of information to understand how biological organisms function and responds to perturbations. It uses a range of computational methods and relies extensively on massive amounts of data provided by high throughput methods and parallel large-scale measurements. A significant and urgent problem of molecular systems biology is the integration of new data into the preexisting, accumulated data, and then re-interpretation of the newly integrated total information. This is particularly acute in transcriptomics, where microarrays and RNA sequencing generate and daily append large amount of new data into the established databanks. Individual researchers and groups can relatively easily, although not inexpensively, produce and submit new transcriptomics data, but are often stymied trying to incorporate their contributions into the established, preexisting knowledge. Information technology core facilities, on the other hand, while providing data storage and access to analysis programs, seldom understand the specifics of individual investigator’s research areas and such cores usually find it difficult to customize their analysis for a particular, distinct project.

Here, we use readily available, web-based, free computational metaanalysis methods to integrate the transcriptional consequences of treating human epidermal keratinocytes with EGF with the related, existing transcriptomics data in public databanks.

EGF was among the first signalling molecules isolated, and was named for its capacity to accelerate epidermal differentiation and eye opening in newborn mice [1]. EGF has become a paradigm for studies of extracellular and intracellular signalling [2-5]. EGF binds to its receptor, EGFR, causing receptor dimerization and consequent activation of its cytoplasmic kinase [6]. The activated kinase initiates several signal transduction cascades of protein phosphorylation, resulting in activation of transcription factors that regulate expression of many genes [7]. EGF promotes cell survival, proliferation, chemotactic migration, and suppresses terminal differentiation, inhibits apoptosis etc. [8]. EGFR is a member of the ErbB protein family and binds several ligands in addition to EGF, e.g., TGFα, and HB-EGF [9].

The transcriptome changes caused by EGF have not been extensively explored in its eponymous tissue, although EGF is very important in the healthy epidermal homeostasis and in several diseases [10]. Specifically in the epidermis, EGF is known to contribute to wound healing, regulate barrier function, suppress terminal differentiation, cause loss of adhesion, induce secreted proteases etc. [10,11]. EGFR is over-expressed in squamous cell carcinomas [12]. Perhaps understandably, the published microarray analyses of the effects of EGF mainly focused on breast cancer and glioblastoma cell lines, the two types of cancer with EGFR-associated aetiology and have provided valuable data on the molecular effect of its action in several cell lines [13-15]. Detailed molecular understanding of the EGFR kinase domain led to development of specific inhibitors, Gefitinib, Erlotinib, Herceptin, Cetuximab, currently used to treat breast, lung, ovarian, prostate, head-and-neck and other cancers [8,16].

To define systematically and comprehensively the transcriptional changes caused by EGF in primary human epidermal keratinocytes, we treated these cells with EGF and compared parallel treated and control cultures using Affymetrix microarrays. We then used metaanalysis programs to integrate the observed changes with a large set of already existing data on transcriptional profiling in epidermal keratinocytes and with data of EGF-treated non-epidermal cell types in GEO database. We found that EGF affects keratinocyte proliferation, attachment and motility and, surprisingly induces DUSPs, phosphatases that attenuate the EGF signal transduction in a feed-back loop. Metaanalysis identified specific overlapping effects of EGF with those of IL-1 and IFNγ, which play a role in keratinocyte activation, inflammation and wound healing. Conversely, we identified the specific genes and processes suppressed by EGF, but induced by pro-differentiation agents. Metaanalysis comparison of EGF effects in other cell types identified extensive similarities between the responses in keratinocytes and in other epithelial cell types, but differences with e.g., endothelial cells. We also found characteristic differences with transformed, oncogenic epithelial cell lines. Our approach can serve as a paradigm for integration of new omics data into preexisting databases and analysis of the integrated data.

## Results

### Early time points, 1 and 4 hrs

Approximately 520 genes were induced by 50% or more at any of the time points, and 580 were suppressed by the EGF treatment in human epidermal keratinocytes. The regulated genes are listed in the Additional file 1: Table S1. A small number of genes, 44, are both induced and suppressed by EGF, at different time points; of these, 34 are induced early, but suppressed late and 10 have just the opposite regulation pattern.

Intriguingly, among the most prominent induced genes are 5 dual specificity phosphatases, DUSP-1, -2, -4, -5 and −6 (Table 1 and Additional file 2: Table S3a, Additional file 3: Table S4a). These enzymes de-phosphorylate, and thereby inactivate, the kinases of the ERK pathways [17,18]. As a result of this de-phosphorylation, the signal transduction cascades activated by the EGF are attenuated. Apparently, the induction of the DUSPs is one of the important feed-back mechanisms that limits the intensity and duration of the EGF effects in keratinocytes.

**Table 1:** Categories over-represented among the EGF regulated genes

**Term**	**Count**	**PValue**
**1 h UP**		
Dual specificity protein phosphatase	5	7.42E-08
MAP kinase phosphatase	5	4.38E-07
regulation of cell proliferation	22	1.03E-06
MAP kinase tyrosine/serine/threonine phosphatase activity	5	2.34E-06
regulation of transcription from RNA polymerase II promoter	20	4.92E-06
Cell cycle control	14	6.27E-06
regulation of apoptosis	20	2.05E-05
Rhodanese-like	5	2.06E-05
nuclear lumen	24	2.14E-05
Cell proliferation and differentiation	21	2.19E-05
Basic-leucine zipper (bZIP) transcription factor	6	3.16E-05
organelle lumen	27	3.32E-05
vasculature development	11	3.44E-05
response to organic substance	18	6.09E-05
JNK cascade	6	8.69E-05
**4 h UP**		
regulation of cell proliferation	46	4.29E-15
regulation of cell motion	22	1.30E-12
plasma membrane part	68	8.83E-12
cell adhesion	25	1.25E-10
vasculature development	22	1.97E-10
plasma membrane	91	2.71E-10
Focal adhesion	23	2.71E-10
extracellular region part	40	2.87E-10
signal	75	1.24E-09
basement membrane	12	6.78E-09
response to wounding	29	6.99E-09
Signal transduction	85	7.80E-09
integrin binding	11	9.78E-09
**24 h UP**		
Dual specificity protein phosphatase	4	1.07E-05
Secreted	26	2.04E-05
wound healing	10	2.17E-05
angiogenesis	9	2.37E-05
ectoderm development	10	3.00E-05
disulfide bond	36	3.23E-05
EGF-like region, conserved site	11	3.30E-05
MAP kinase phosphatase	4	3.62E-05
heparin-binding	6	4.53E-05
regulation of cell migration	9	6.13E-05
extracellular region part	19	7.79E-05
Skin	26	1.11E-04
epidermis development	9	1.11E-04
signal	37	1.25E-04
MAP kinase tyrosine/serine/threonine phosphatase activity	4	1.43E-04
regulation of cell proliferation	18	1.57E-04
Pathways in cancer	13	1.79E-04
**48 h UP**		
Metallothionein	8	1.21E-12
acetylated amino end	13	2.39E-11
16q13	8	5.32E-10
acetylation	47	1.11E-07
Keratinocyte	11	1.35E-07
response to protein stimulus	9	1.08E-05
peptide cross-linking	5	1.16E-04
Cajal-Retzius cell	9	2.25E-04
Chaperone	8	2.75E-04
Steroid biosynthesis	5	2.81E-04
1q21	5	3.43E-04
Kidney	26	5.22E-04
abdominal aortic aneurysm	5	8.75E-04
**1 h DOWN**		
glycoprotein	81	8.14E-11
signal	67	2.24E-10
plasma membrane part	56	1.11E-09
disulfide bond	59	1.15E-08
Secreted	37	2.93E-06
calcium binding	8	5.58E-05
extracellular region part	26	5.75E-05
regulation of cell motion	11	1.41E-04
Cell adhesion	19	1.62E-04
defense response	20	2.11E-04
calcium	20	2.27E-04
Keratinocyte	8	2.57E-04
sialic acid	4	4.26E-04
disease mutation	30	4.71E-04
Cell junction protein	7	7.65E-04
response to wounding	17	8.70E-04
apical part of cell	9	9.02E-04
**4 h DOWN**		
Ichthyosis	7	3.02E-08
epidermis development	12	2.90E-07
epithelial cell differentiation	9	1.55E-05
cell cycle	12	1.71E-04
Keratinocyte	7	1.95E-04
Metabolism of xenobiotics by cytochrome P450	6	2.24E-04
epidermal cell differentiation	6	2.94E-04
Foreskin	5	4.58E-04
microsome	6	5.75E-04
S100/CaBP-9 k-type, calcium binding	4	9.73E-04
disease mutation	22	9.98E-04
**24 h DOWN**		
Intermediate filament	7	2.11E-06
Insulin-like growth factor binding protein, N-terminal	4	3.13E-05
disease mutation	21	6.00E-05
Thyroglobulin type-1	4	9.49E-05
Structural protein	9	1.06E-04
response to abiotic stimulus	11	1.11E-04
epidermis development	8	1.67E-04
Aldehyde dehydrogenase NAD(P)-dependent	3	1.68E-04
desmosome	4	1.82E-04
response to steroid hormone stimulus	8	2.17E-04
signal	30	4.98E-04
Desmosomal cadherin	3	5.82E-04
response to organic substance	14	5.96E-04
keratin	6	6.72E-04
**48 h DOWN**		
phosphoprotein	120	2.06E-10
response to organic substance	33	1.73E-09
response to endogenous stimulus	24	4.75E-09
regulation of programmed cell death	34	8.33E-09
Cell cycle control	23	1.43E-08
response to steroid hormone stimulus	16	4.35E-08
negative regulation of apoptosis	21	5.63E-08
response to hormone stimulus	21	1.02E-07
apicolateral plasma membrane	11	2.74E-07
Desmosomal cadherin	5	5.95E-07
response to wounding	23	2.37E-06
acetylation	52	5.00E-06
response to extracellular stimulus	14	8.15E-06
response to glucocorticoid stimulus	9	9.28E-06
regulation of cell growth	13	1.16E-05
response to nutrient	11	1.83E-05

Selected categories from DAVID analysis are presented, sorted from the best p-values from each set. UP denotes induced by EGF, DOWN suppressed. Count gives the number of regulated genes in each category.

As expected, EGF, being a fundamental and wide-reaching signal, affects a fairly large number of signalling pathways. Immediately after addition, i.e., in the first hour, EGF suppressed production of 67 genes in the ‘signalling’ ontological category, including 3 insulin-like growth factor binding proteins, mucins, cytokines and their receptors as well as secreted lectins, enzymes with signalling function, such as kallikrein peptidases, and structural proteins with signalling function, such as cadherins, desmocollins and desmogleins. Presumably, their suppression inactivates the corresponding active signalling pathways, and prepares the cells for new biological processes and reactions. At the 4 h time point, 75 signalling genes are induced, again comprising a wide range of functions and pathways. For example, proteases with signalling function, ADAM8, ADAM22, cathepsins, thrombospondin, coagulation factors and MMPs are induced, but so are protease inhibitors, such as serpins, TIMP1 etc. Also induced at this time point are integrins and components of the extracellular matrix, e.g., Laminin V and tenascin. Particularly noteworthy is the induction of amphiregulin and TGFα (Additional file 2: Table S3b); both are EGFR activating ligands that can sustain or extend the initial signal by EGF [19,20].

Among the regulators of proliferation and cell cycle, which are induced after 1 h of EGF treatment, we find CDK7, the protein thought to serve as a direct link between the regulation of transcription and the cell cycle [21,22]. Transcription factors including BTG2, cyclin G2, GADDα, Jun, Jun B, v-fos etc. are also induced (Additional file 2: Table S3c). Interestingly, regulation of apoptosis is also one of the ontological categories induced early after EGF treatment (Additional file 2: Table S3d). Parenthetically, we note that the negative regulators of apoptosis are suppressed at the 48 h time point (see below, Additional file 3: Table S4k).

EGF strongly affects cell adhesion and motility through induction of focal adhesion, basement membrane and integrin binding protein categories. Cell adhesion and motility are complex processes that require a large set of coordinated protein functions; EGF affects these processes by inducing certain genes, e.g., α-actinin, cadherin 13, thrombin receptor, SMAD3 and SMAD7, VEGF-A and VEGF-C (Additional file 2: Table S3b, 3e), while suppressing others, e.g., desmocollins 1 and 2, desmoglein 1, cadherins, etc. (Additional file 2: Table S3h). In addition, EGF induced the expression of several proteins that, when mutated, cause in *Epidermolysis bullosa*, such as Integrin α2, laminin α3, (Additional file 2: Table S3e). It is unclear at present whether EGF-targeted treatment may be beneficial in certain *Epidermolysis bullosa* patients.

**Figure 1:**
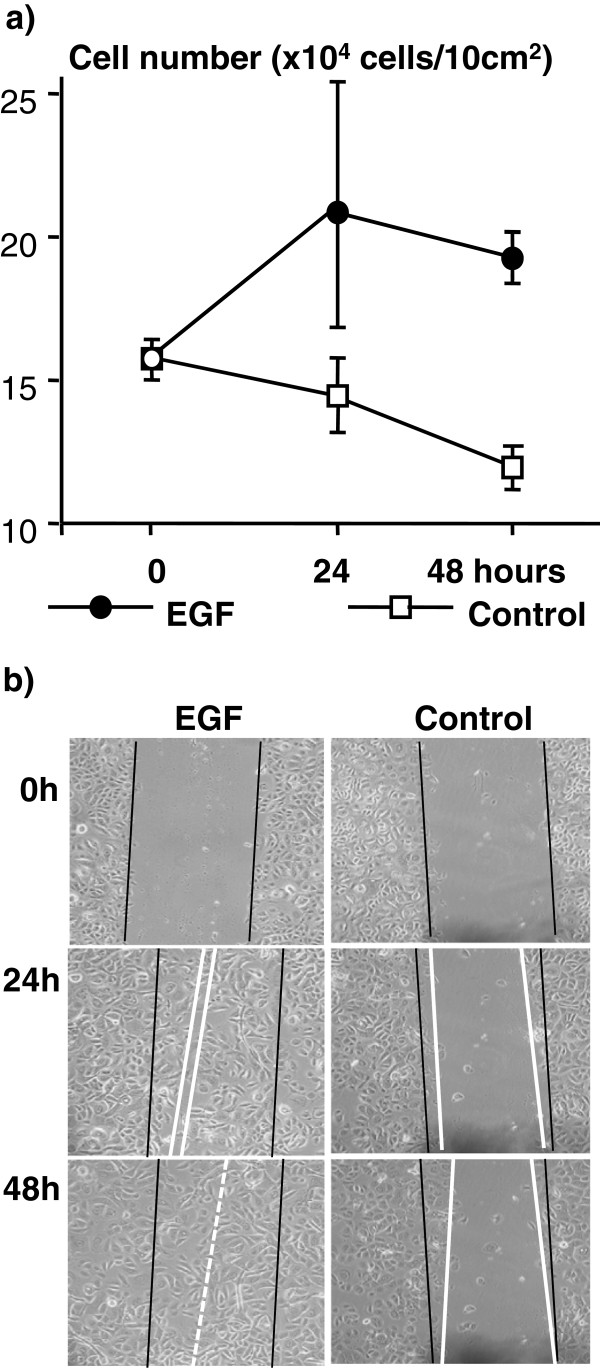
**EGF promotes keratinocyte proliferation and migration. a)** Cell counts in the EGF-treated and EGF-starved cultures. The results are derived from triplicate experiments. **b)** The scratch assay. The black lines mark the initial boundaries of the scratches, the white lines the leading fronts of the migrating cells.

To confirm the proliferative and pro-migratory effects of EGF in human epidermal keratinocytes, we counted the cells in EGF-treated and control cultures, and we examined the re-epithelialization of scratches in culture (Figure 1). As expected from previous studies and from the results described above, the addition of EGF profoundly improved both the proliferation and migration of keratinocytes.

Importantly, EGF induces defence and wounding response genes in keratinocytes. These include angiogenic signalling proteins, such as VEGF-A, VEGF-C, PTK2B, activin A receptor, cadherin 13, CD59 and CD55, placental growth factor, plasminogen activator, urokinase, TGFα, (Additional file 2: Table S3f). Additionally, proinflammatory cytokines, including IL-1b and IL-6, are induced. The induction of defence and wounding response genes by EGF is congruent with the known protective role of EGF during skin wound healing [19]. Curiously, certain genes in the ontological categories of defence and wounding responses, such as fibrinogen α, PDGF receptor, lysozyme etc., are simultaneously suppressed by EGF (Additional file 2: Table S3g). However, in general, the function of the suppressed genes in the defence and wounding responses ontological categories is different from the function of the induced ones: the suppressed genes often include immune system modulators, e.g., IL-4, and interferon-α5, as well as the infection fighting proteins e.g., defensin b-1, lysozyme, TLR1, S100A8, etc. The ontological category of wounding response is broad and apparently EGF promotes certain aspects thereof, such as re-epithelialization and wound closure, while repressing others, such as antimicrobial processes and certain aspects of the immune response.

Interestingly, many proteins known as markers of keratinocyte differentiation are suppressed by EGF (Additional file 2: Table S3i). These include keratins KRT1 and KRT10, filaggrin, desmosomal proteins, SPRR and also members of the Ca-binding, S100 family genes.

### Late time points, 24 and 48 hrs

Importantly, the DUSPs remain induced in the EGF-treated keratinocytes at the late time points as well, specifically DUSP-4, -5, -6 and −7 (Additional file 3: Table S4a). At 24 h EGF induces expression of several secreted proteins, such as proteases, including MMP1, 9 and 10, as well as protease inhibitors, such as serpins; presumably, these play a role in matrix remodelling, given that matrix components fibronectin and laminin are also induced (Additional file 3: Table S4b). Among the secreted growth factors we particularly note TGFα and HB-EGF, the EGFR ligands, feed-back extenders of the original signal. We also note the induction of VEGF-A and VEGF-C, two angiogenic factors (see below).

Genes promoting proliferation are induced at 24 h, including HB-EGF and TGFα, as well as additional secreted signals and their receptors (Additional file 3: Table S4c). Wound healing and angiogenesis proteins are induced, including CD73, ADAM8, DKK1, endothelin receptor A, fibronectin 1, integrin α2, interferon-γ receptor 2, MMP1, 9 and 10 urokinase, urokinase receptor, plasminogen activator and serpins-B2 and -E2, (Additional file 3: Table S4d). Extracellular matrix structural and remodelling proteins TIMP1, annexin A2, fibronectin 1, laminin γ2, MMPs etc. are induced after 48 h (Additional file 3: Table S4d).

Interestingly, and in contrast with the early time points, certain keratinocyte-specific genes are induced by EGF, including some markers of keratinocyte differentiation. These include S100 calcium binding proteins, SPRRs, involucrin, serpin-B1 etc. (Additional file 3: Table S4e). Lipid and steroid biosynthesis enzymes, tangential markers of epidermal differentiation, are also induced; these include ATP citrate lyase, sterol isomerase, hydroxysteroid-17-β-dehydrogenase 2 and pyruvate carboxylase. Unexpectedly, 48 h after EGF addition, 8 different metallothioneins are induced (Additional file 3: Table S4f); their role in keratinocytes in response to EGF is unknown.

The ontological categories suppressed at 24 and 48 hrs include cell cycle and apoptosis. The cell cycle regulation 48 h after EGF addition continues primarily by suppression of cyclins A2, B1, B2 and CDK6, although notably, CDK inhibitors p19, p21 and p57 are also suppressed (Additional file 3: Table S4k). Desmosome components and cadherins are suppressed at 24 and 48 hrs, as are several keratin genes (Additional file 3: Table S4h). Unexpectedly, we find KRT16 in the suppressed gene set; KRT16 is a known marker of proliferation and is induced in the early times after EGF addition [23]. The suppression of these cytoskeletal proteins may play a role in keratinocyte adhesion and locomotion.

A set of genes that respond to steroids is suppressed by EGF at the late time points (Additional file 3: Table S4i). This we find curious, because, as noted above, the steroid biosynthesis enzymes are induced (Additional file 2: Table S3e). Perhaps the locally produced steroids accumulate at an even later time point. Alternatively, these steroid-responsive genes are suppressed specifically to modulate the steroid response; this interesting metabolic issue deserves further examination.

### Transcription factors

The transcriptional changes in response to EGF are, presumably, effected by changes in the activity of transcription factors (TF). To identify the putative transcription factors responsible, we searched for TF binding sites statistically overrepresented in the regulated genes using DAVID [24]. Interestingly, we find that the induced genes contain an overabundance of BACH1 and BACH2 binding sites (Table 2a). Statistically, the BACH binding sites are the most prominent ones in the regulated genes at all time points. The BACH proteins are basic leucine zipper transcription factors, can function both as transcription activators and as transcriptional repressors and regulate transcription of genes involved in G1/S and G2/M phases of the cell cycle; they form heterodimers with MafK proteins and are known to be sensitive to oxidative stress [25,26]. Inhibition of BACH1 reduces UV light-caused damage in keratinocytes, apparently through regulating the expression of heme oxygenases [27]. *In vitro*, BACHs bind to AP1/NF-E2 binding sites, TGA(GC)TCA [28]. We note that transcriptionally functional AP1 sites have been found in a large proportion of keratinocyte differentiation marker genes [29-33]. The expression of the individual members of the AP1 protein family in different layers of the epidermis is controversial. It is, therefore, possible that the BACH proteins perform functions until now attributed to the AP1 proteins. Our results suggest that the BACH proteins play important roles in keratinocyte proliferation and differentiation, in particular in response to EGF.

**Table 2:** Transcription factor binding sites over-represented in the promoters of the EGF regulated genes

					
**a**					
**Name**	**Count**	**%**	**PValue**	**Benjamini**	**FDR**
**1 h Up**					
**BACH2	65	53	**5.60E-05**	9.80E-03	6.86E-02
**IK3**	65	53	**1.48E-04**	1.29E-02	1.81E-01
**LMO2COM**	80	65	**1.93E-04**	1.12E-02	2.36E-01
**PAX3**	63	51	**3.25E-04**	1.42E-02	3.98E-01
**TATA**	74	60	**3.34E-04**	1.17E-02	4.10E-01
**BACH1	70	57	**3.52E-04**	1.03E-02	4.31E-01
**CEBPB**	75	61	**4.51E-04**	1.13E-02	5.52E-01
**AP1	80	65	**4.54E-04**	9.94E-03	5.56E-01
**SOX5**	67	54	**5.35E-04**	1.04E-02	6.54E-01
**NFE2	53	43	**6.09E-04**	1.07E-02	7.45E-01
**4 h Up**					
**BACH2	142	63	**2.35E-17**	4.13E-15	2.88E-14
**AP1	172	76	**3.74E-16**	2.93E-14	4.11E-13
**BACH1	152	67	**2.67E-15**	1.56E-13	3.28E-12
**IK3**	127	56	**1.18E-09**	5.21E-08	1.45E-06
**STAT**	106	47	**5.97E-09**	2.10E-07	7.33E-06
**SRY**	119	52	**1.19E-08**	3.49E-07	1.46E-05
**NFE2	105	46	**1.85E-08**	4.66E-07	2.28E-05
**TATA**	142	63	**2.52E-08**	5.53E-07	3.09E-05
**FOXO1**	116	51	**4.89E-08**	9.56E-07	6.00E-05
**LMO2COM**	148	65	**3.69E-07**	6.49E-06	4.52E-04
**FREAC3**	115	51	**7.41E-07**	1.19E-05	9.09E-04
**RORA2**	124	55	**8.78E-07**	1.29E-05	1.08E-03
**TAXCREB**	137	60	**1.30E-06**	1.76E-05	1.59E-03
**FOXO4**	137	60	**1.39E-06**	1.75E-05	1.70E-03
**HLF**	109	48	**1.70E-06**	2.00E-05	2.09E-03
**CHOP**	124	55	**2.61E-06**	2.87E-05	3.20E-03
**MYOD**	139	61	**3.76E-06**	3.90E-05	4.62E-03
**NFAT**	110	48	**3.80E-06**	3.72E-05	4.67E-03
**FREAC7**	124	55	**4.02E-06**	3.72E-05	4.93E-03
**HFH1**	117	52	**4.16E-06**	3.66E-05	5.10E-03
**MEF2**	173	76	**5.42E-06**	4.54E-05	6.65E-03
**MEIS1BHOXA9**	127	56	**6.44E-06**	5.15E-05	7.90E-03
**POU3F2**	144	63	**6.77E-06**	5.18E-05	8.30E-03
**FREAC4**	118	52	**8.04E-06**	5.90E-05	9.87E-03
**TST1**	114	50	**8.68E-06**	6.11E-05	1.07E-02
**CREBP1**	120	53	**1.05E-05**	7.09E-05	1.29E-02
**FOXO3**	79	35	**1.23E-05**	8.04E-05	1.51E-02
**SOX5**	121	53	**1.47E-05**	9.26E-05	1.81E-02
**HSF2**	108	48	**1.96E-05**	1.19E-04	2.41E-02
**LYF1**	102	45	**2.33E-05**	1.37E-04	2.86E-02
**RSRFC4**	118	52	**2.58E-05**	1.46E-04	3.16E-02
**FREAC2**	88	39	**2.62E-05**	1.44E-04	3.21E-02
**CEBPA**	80	35	**2.66E-05**	1.42E-04	3.27E-02
**IK2**	83	37	**2.78E-05**	1.44E-04	3.41E-02
**CETS1P54**	72	32	**3.21E-05**	1.62E-04	3.94E-02
**b**					
**Term**	**Count**	**%**	**PValue**	**Benjamini**	**FDR**
**1 h Down**					
**SOX5**	94	48	**1.47E-03**	2.28E-01	1.79E + 00
**CEBPB**	104	54	**3.43E-03**	2.61E-01	4.13E + 00
**STAT**	71	37	**3.75E-03**	1.98E-01	4.51E + 00
**POU3F2**	109	56	**5.57E-03**	2.18E-01	6.62E + 00
**FOXO3**	57	29	**6.14E-03**	1.95E-01	7.28E + 00
**AP1	107	*	2.33E-02		
**4 h Down**					
**HLF**	59	47	**7.35E-04**	1.21E-01	8.98E-01
**POU3F2**	77	62	**3.05E-03**	2.36E-01	3.68E + 00
**NCX**	61	49	**4.17E-03**	2.18E-01	5.00E + 00
**TATA**	70	56	**6.68E-03**	2.55E-01	7.90E + 00
**E4BP4**	58	46	**8.38E-03**	2.56E-01	9.82E + 00
**CEBPB**	71	57	**8.52E-03**	2.22E-01	9.96E + 00
**AP1	73	*	3.57E-02		
**24 h Down**					
**BACH2	47	48	**5.35E-03**	6.11E-01	6.38E + 00
**AP1	58	*	2.85E-02		
**48 h Down**					
**HLF**	92	45	**2.37E-04**	4.10E-02	2.90E-01
**LMO2COM**	125	62	**2.59E-04**	2.26E-02	3.17E-01
**MEF2**	151	74	**3.56E-04**	2.08E-02	4.36E-01
**FOXJ2**	136	67	**3.86E-04**	1.70E-02	4.74E-01
**SOX9**	98	48	**4.04E-04**	1.42E-02	4.96E-01
**CEBPB**	117	58	**6.03E-04**	1.76E-02	7.38E-01
**AP1	125	62	**7.64E-04**	1.91E-02	9.34E-01
**TATA**	114	56	**8.00E-04**	1.75E-02	9.78E-01
**HFH3**	89	44	**1.44E-03**	2.80E-02	1.76E + 00
**IRF7**	97	48	**1.49E-03**	2.61E-02	1.82E + 00
**IRF2**	89	44	**1.66E-03**	2.64E-02	2.02E + 00
**NFAT**	89	44	**2.78E-03**	4.03E-02	3.37E + 00
**TAXCREB**	111	55	**3.84E-03**	5.10E-02	4.61E + 00
**HAND1E47**	97	48	**4.11E-03**	5.08E-02	4.94E + 00
**E4BP4**	91	45	**4.99E-03**	5.73E-02	5.96E + 00
**FREAC2**	71	35	**5.12E-03**	5.52E-02	6.11E + 00
**POU3F2**	119	59	**5.18E-03**	5.26E-02	6.18E + 00
**STAT1**	84	41	**5.88E-03**	5.63E-02	6.99E + 00

a) Induced genes, b) suppressed genes. The top p-value sites are presented for each time point. Count columns give the number of genes with the respective binding sites, % the percentage of submitted genes that have the site, while Benjamini and FDR (false discovery rate), represent statistical values corrected for multiple comparisons. Note the recurrence of BACH1, BACH2, AP1 and NF-E2 sites, marked with double asterisk, which bind to similar DNA sequences. At 4 and 24 h, in suppressed genes, the AP1 sites barely miss the statistical cut off p-value of 0.01; they are included here nevertheless.

The genes induced at early time points additionally contain a statistical overabundance of IK3, LMO2COM, PAX3 and SOX5 binding sites. While these results suggest that these four TFs mediate the EGF response, it is also possible that the overrepresented consensus binding sites could be recognized by other, related transcription factors in epidermal keratinocytes.

Much fewer TF binding sites reached statistical significance in the promoters of suppressed genes (Table 2b). We note that CEBPβ sites have been identified at both early and late time point genes. CEBPβ regulates genes involved in immune and inflammatory responses and has been shown to bind to regulatory regions of several acute-phase and cytokine genes [34-36]. Additionally, binding sites for POU3F2 (a.k.a. BRN2, OCT7) appear over-represented in the suppressed genes. This transcription factor has not been noted in keratinocytes before (although it is important in melanocytes and melanoma [37]).

As a final point, we note that AP1 sites are common in the suppressed genes as well (marked with asterisks in Table 2), although only at 48 hrs do the AP1 sites reach our significance cut-off p-value better than 0.01. Clearly, the relative contributions of BACH, AP1 and NF-E2 transcription factors in epidermal keratinocytes, their differentiation and their responses to EGF require more careful, in-depth study.

### Metaanalysis, part 1: Intersections of genes regulated by EGF and by other extracellular agents in keratinocytes

**Figure 2:**
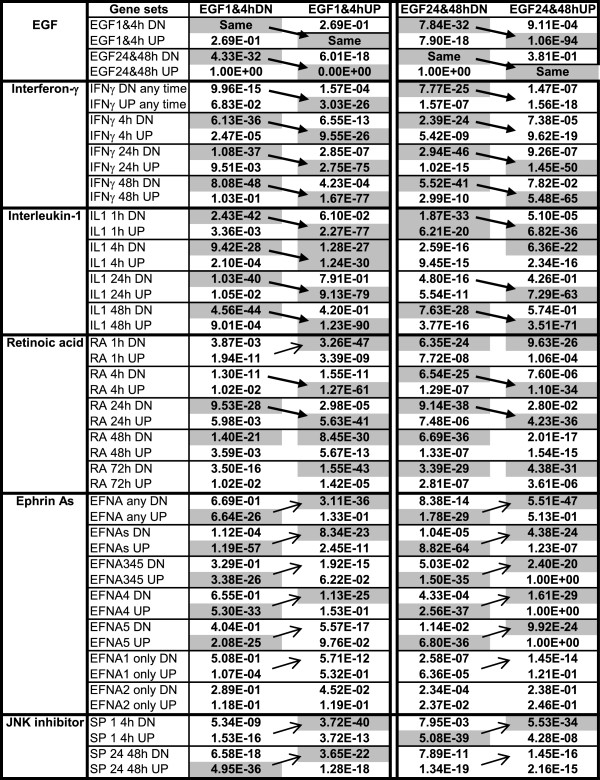
**Overlaps of the lists of genes regulated by EGF with those regulated by other extracellular agents.** We show overlaps with genes regulate by Interferon-γ, IL-1, Retinoic acid, the five EphrinA proteins and the JNK inhibitor SP600125. UP denotes induced, DN suppressed genes. The overlaps with p-value better than 10^-20^ are marked with gray. The downward arrows mark the high overlaps between both the induced and the suppressed gene lists. The upward arrows mark the antiparallel overlaps, i.e., between the lists of genes that are induced by EGF, but are suppressed by other agents, and *vice versa*.

We compared the lists of genes regulated by EGF with lists of genes identified previously as regulated by extracellular signals, namely Interferon-γ (IFNγ), Interleukin 1 (IL-1), Retinoic acid (RA), Ephrin As and SP600125, a specific inhibitor of JNK [38-42]. The numbers of genes present in each list analyzed are given in Additional file 4: Table S5. We used the Lists2Networks program for comparisons and collected both the matrix of p-values for overlapping of related lists, as well as Bonferoni-corrected lists of gene ontology categories ‘biological process’ and ‘molecular function’ [43]. We also examined GenMAPP and KEGG pathways, KEA kinase targets, predicted promoter sites and OMIM disease-associated genes. The gene ontology biological process comparisons gave the most complete and detailed results and these will be presented here (Figure 2, Tables 3 and 4); the other categories gave qualitatively similar, if sparser results (not shown).

**Table 3:** Biological processes common in the EGF-, Interferon-γ- and IL-1-regulated genes

		
**a**		
** Gene ontology: Biological process**	**EGF1&4hUP**	**IFN4hUP**
biological_process	5.84E-07	6.38E-06
signal transduction	3.62E-05	1.59E-04
response to stress	6.39E-05	1.37E-04
regulation of gene expression	2.27E-05	1.82E-03
regulation of apoptosis	8.12E-04	1.07E-03
cell communication	4.12E-05	5.65E-03
regulation of RNA metabolic process	8.79E-03	4.84E-03
regulation of transcription	7.87E-05	1.50E-02
regulation of transcription,	1.33E-02	9.08E-03
protein metabolic process	5.42E-02	2.34E-05
response to wounding	8.17E-04	6.57E-02
	**EGF24&48hUP**	**IFN48hUP**
biological_process	1.32E-02	3.32E-16
metabolic process	3.16E-02	8.99E-14
** Gene ontology: Biological process**	**EGF1&4hDN**	**IFN4hDN**
response to external stimulus	5.47E-04	1.79E-09
tissue development	6.50E-04	3.64E-05
cell differentiation	6.90E-04	5.94E-10
keratinocyte differentiation	1.42E-02	5.37E-04
epidermis development	8.64E-06	1.49E-02
response to chemical stimulus	2.22E-02	4.54E-08
defense response	2.05E-02	2.31E-03
response to stress	2.32E-02	2.67E-08
	**EGF24&48hDN**	**IFN48hDN**
response to stress	1.95E-06	9.50E-10
response to external stimulus	5.54E-04	4.70E-03
regulation of cell proliferation	2.87E-03	3.97E-03
biological_process	1.45E-02	3.66E-24
regulation of cell cycle	7.16E-02	1.01E-04
tissue development	7.13E-02	4.06E-02
**b**		
** Gene ontology: Biological process**	**EGF1&4hUP**	**IL1&1hUP**
regulation of cell proliferation	1.65E-07	4.09E-08
biological_process	5.84E-07	3.75E-12
regulation of signal transduction	1.41E-07	2.34E-06
regulation of gene expression	2.27E-05	2.84E-12
signal transduction	3.62E-05	1.16E-06
cell communication	4.12E-05	4.90E-06
response to stress	6.39E-05	7.05E-11
regulation of transcription	7.87E-05	1.60E-12
regulation of cell motion	1.45E-07	9.43E-05
negative regulation of cell proliferation	5.55E-05	6.13E-05
negative regulation of prog. cell death	6.27E-04	5.05E-05
regulation of apoptosis	8.12E-04	3.33E-09
response to wounding	8.17E-04	7.63E-05
regulation of cell migration	6.33E-05	9.95E-04
positive regulation of chemotaxis	2.38E-04	1.68E-03
negative regulation of apoptos	3.07E-03	1.73E-04
positive regulation of signal	3.47E-03	1.15E-05
positive regulation of metabolism	4.70E-03	3.42E-08
positive regulation of cell process	1.64E-03	3.11E-03
regulation of RNA metabolic process	8.79E-03	1.06E-10
phosphate metabolic process	1.08E-02	1.26E-05
regulation of transcription,	1.33E-02	6.42E-11
response to external stimulus	2.35E-03	1.33E-02
regulation of transcription	1.59E-02	6.90E-10
transmembrane receptor protein	1.49E-02	1.59E-03
positive regulation of gene expression	3.33E-02	4.02E-06
positive regulation of cell motility	6.15E-04	3.31E-02
protein metabolic process	5.42E-02	1.31E-06
regulation of catalytic activity	7.77E-02	1.89E-08
transforming growth factor beta pathway	8.47E-02	6.35E-03
positive regulation of transcription	9.37E-02	1.88E-06
** Gene ontology: Biological process**	**EGF24&48hUP**	**IL1&48hUP**
biological_process	1.32E-02	8.89E-19
metabolic process	3.16E-02	1.32E-16
	**EGF1&4hDN**	**IL1 1hDN**
cell differentiation	6.90E-04	3.12E-03
epidermis development	8.64E-06	1.60E-02
keratinocyte differentiation	1.42E-02	3.78E-03
	**EGF24&48hDN**	**IL1 48hDN**
epidermis development	5.18E-03	6.21E-03
biological_process	1.45E-02	1.75E-14
response to stress	1.95E-06	3.26E-02
response to external stimulus	5.54E-04	4.08E-02
	**EGF1_4hUP**	**EFNAs_DN**
response to stress	6.39E-05	3.67E-02
	**EGF24_48hUP**	**EFNAs_DN**
biological_process	1.32E-02	6.92E-03
metabolic process	3.16E-02	3.29E-02
	**EGF1_4hUP**	**RA4hDN**
regulation of gene expression	2.27E-05	8.66E-05
regulation of transcription	7.87E-05	2.69E-04
cell communication	4.12E-05	2.62E-03
biological_process	5.84E-07	5.66E-03
signal transduction	3.62E-05	6.86E-03
regulation of RNA metabolic process	8.79E-03	8.62E-05
regulation of cell migration	6.33E-05	1.03E-02
regulation of apoptosis	8.12E-04	1.01E-02
regulation of transcription	1.33E-02	1.88E-04
regulation of transcription	1.59E-02	6.81E-04
regulation of cell motion	1.45E-07	2.60E-02
regulation of cell proliferation	1.65E-07	4.18E-02
positive regulation of metabolism	4.70E-03	4.57E-02
regulation of signal transduction	1.41E-07	5.12E-02
	**EGF1_4hDN**	**EFNAs_UP**
epidermis development	8.64E-06	9.39E-09
tissue development	6.50E-04	1.52E-05
cell differentiation	6.90E-04	1.80E-07
keratinocyte differentiation	1.42E-02	1.58E-11
	**EGF24_48hDN**	**EFNAs_UP**
epidermis development	5.18E-03	9.39E-09
biological_process	1.45E-02	5.83E-04
tissue development	7.13E-02	1.52E-05
	**EGF1_4hUP**	**RA4hUP**
biological_process	5.84E-07	7.73E-05
regulation of gene expression	2.27E-05	1.05E-04
regulation of transcription	7.87E-05	2.86E-04
signal transduction	3.62E-05	3.42E-04
regulation of signal transduction	1.41E-07	4.46E-04
response to stress	6.39E-05	8.06E-04
negative regulation of program	6.27E-04	4.51E-04
cell communication	4.12E-05	2.77E-03
negative regulation of apoptosis	3.07E-03	3.61E-04
positive regulation of signal	3.47E-03	4.51E-04
phosphate metabolic process	1.08E-02	8.40E-05
regulation of RNA metabolic process	8.79E-03	4.78E-03
regulation of transcription,	1.33E-02	6.50E-03
regulation of transcription	1.59E-02	5.92E-03
positive regulation of metabolism	4.70E-03	1.99E-02
regulation of apoptosis	8.12E-04	4.33E-02
response to external stimulus	2.35E-03	4.24E-02
positive regulation of gene expression	3.33E-02	1.35E-02
protein metabolic process	5.42E-02	1.39E-03
positive regulation of transcription	9.37E-02	7.29E-03

a) The EGF and Interferon-γ overlaps b) The EGF and IL-1 overlaps. UP denotes induced, DN suppressed genes. The numbers represent the p-values. While many ontological categories are redundant, closely related or overly general, note that keratinocyte differentiation is suppressed in all three sets, the EGF-, Interferon-γ-, and IL-1-regulated genes. Also note that processes related to cell motility are induced by EGF and IL-1, but not by IFNγ.

**Table 4:** Biological processes common in the EGF-, EphrinAs- and SP600125-regulated genes

**Gene ontology: Biological process**	**EGF 1&4 h UP**	**SP 1&4 h DN**	**EFNAs DN**
biological process	3.94E-07	9.37E-02	6.92E-03
signal transduction	1.65E-05	1.75E-03	1
cell communication	1.94E-05	4.39E-03	5.49E-01
response to stress	6.39E-05	1	3.67E-02
cell motion	3.70E-03	1	3.70E-02
	**EGF 1&4 h DN**	**SP 1&4 h UP**	**EFNAs UP**
epidermis development	8.64E-06	1	9.39E-09
response to external stimulus	5.47E-04	1.69E-06	1
tissue development	6.50E-04	9.01E-02	1.52E-05
cell differentiation	6.90E-04	4.87E-06	1.80E-07
keratinocyte differentiation	1.42E-02	3.14E-01	1.58E-11
defense response	2.05E-02	5.27E-02	1
response to chemical stimulus	2.22E-02	1.72E-13	1.23E-01
response to stress	2.32E-02	1.40E-07	1
	**EGF 24&48 h UP**	**SP 1&4 h DN**	**EFNAs DN**
biological process	1.32E-02	9.37E-02	6.92E-03
metabolic process	3.16E-02	1.00E + 00	3.29E-02
anatomical structure development	4.50E-02	2.20E-01	1
blood coagulation	6.08E-02	3.59E-01	1
	**EGF 24&48 h DN**	**SP 1&4 h UP**	**EFNAs UP**
response to stress	1.95E-06	5.34E-03	1
response to external stimulus	5.54E-04	1.08E-02	1
regulation of cell proliferati	2.87E-03	4.68E-06	1
epidermis development	5.18E-03	1	9.39E-09
response to wounding	8.27E-03	2.60E-02	1
biological process	1.45E-02	1.72E-03	5.83E-04
negative regulation of proliferation	1.56E-02	4.83E-02	1
tissue development	7.13E-02	1	1.52E-05
regulation of apoptosis	8.77E-02	9.18E-01	1

UP denotes induced, DN suppressed genes. Note that the genes induced by EGF are suppressed by EphrinAs and SP600125, and *vice versa*.

Comparing lists of EGF-regulated genes revealed very interesting patterns: EGF, IFNγ and IL-1 induce and suppress related sets of genes, while EphrinAs and SP600125 have antiparallel effects: genes induced by EGF are suppressed by EphrinAs and SP600125, and *vice versa* (Figure 2). Even more intriguing is the comparison of the EGF- and RA-regulated genes: parallel at 1 h after RA addition, the relationship is antiparallel after 4 and 24 h and lost after 48 hrs (Figure 2).

In comparisons of regulated genes, one finds many redundant or overly general ontological categories, which have to be parsed in order to detect the specific and important ontological categories regulated in parallel. For example, comparing lists of EGF- and IFNγ-regulated genes (Table 3a), we find in common very general categories, such as ‘biological process,’ ‘metabolic process’ or ‘cell communication.’ However, we also find specific categories: at early time points both EGF and IFNγ induce common regulators of transcription and, importantly, response to stress and response to wounding. Conversely, both EGF and IFNγ suppress keratinocyte differentiation and epidermis development genes. Another set of stress response genes is suppressed in common by EGF and by IFNγ. At the later time points, 24 and 48 h, proliferation and cell cycle genes are suppressed by both (Table 3a).

A somewhat different picture emerges in the comparisons of the EGF- and IL-1-regulated genes (Table 3b). Like IFNγ, IL-1 induces regulators of transcription and responses to stress and wounding. Importantly, unlike IFNγ, IL-1 also induces sets of genes responsible for cell motility and chemotaxis. Again distinctly from IFNγ, IL-1 induces inhibitors of apoptosis. Similar to IFNγ, IL-1 suppresses keratinocyte differentiation and epidermis development genes. This suppression continues in IL-1 treated keratinocytes longer than in the IFNγ-treated ones, and is still noticeable at 48 h (Table 3b).

**Figure 3:**
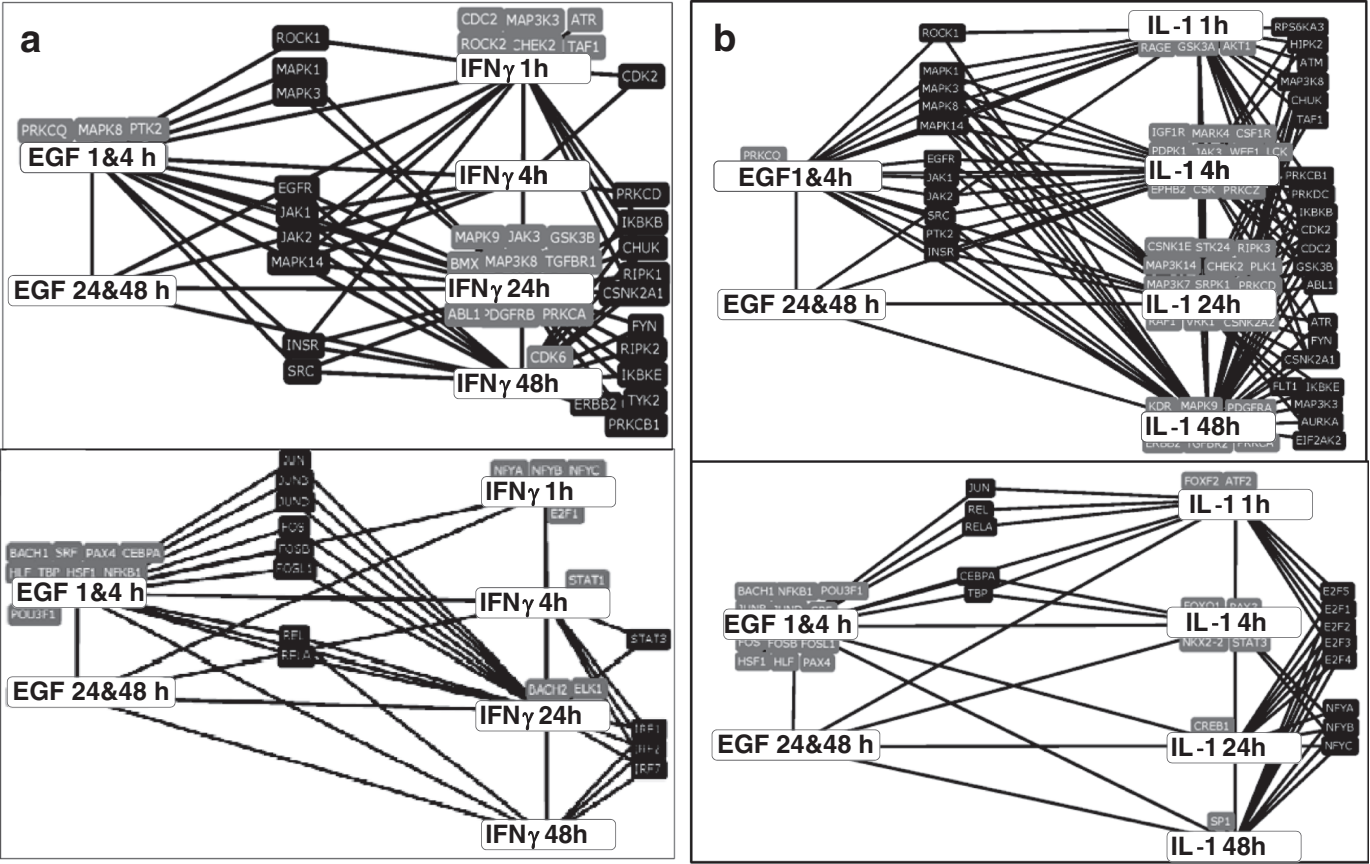
**Overlapping kinase targets and transcription factor target genes. a)** The kinases that target protein products of both the EGF and the Interferon-γ regulated genes are shown on top. The transcription factors that target both the EGF and the Interferon-γ regulated genes are shown on the bottom. **b)** Corresponding kinases and transcription factors for the EGF and IL-1 regulated overlapping targets. Black rounded rectangles denote the kinases and transcription factors associated with multiple time points, gray ones those associated with a single time point. The black rectangles on the right pertain to IFN-γ and IL-1 only, whereas those in the middle bestride the EGF and the IFN-γ/IL-1 sets.

Using the Lists2Networks program [43] for comparisons of predicted transcription factor binding sites in the promoters of regulated genes, we see again characteristic overlaps between the genes co-regulated by EGF and IFNγ and those co-regulated by EGF and IL-1 (Figure 3). For example, both EGF and IFNγ activate many transcription factors of the AP1 family, Fos and Jun, and the Rel NFκB proteins after 24 h (Figure 3a). The overlap of transcription factors targets for EGF and IL-1 is sparser than for EGF and IFNγ; in particular, fewer common AP1 protein binding sites were identified in the genes regulated by both EGF and IL-1.

We also used Lists2Networks to identify the kinases whose targets are overrepresented in the regulated genes [43]. Using the KEA kinase targets analysis, we found that both EGF and IFNγ induce targets of MAPK-1, -3 and −14, as well as of the receptor kinases EGFR, INSR, JAK-1 and −2. However, only ROCK1 targets are induced already at 1 h, the targets for other kinases are overrepresented in parallel at later times (Figure 3b). EGF, IFNγ and IL-1 induce a similar family of kinase targets, but this induction occurs earlier in the IL-1-treated cells than in the IFNγ-treated ones (Figure 3b). Despite larger sets of common kinase targets induced by EGF and IL-1, the set of transcription factors activated in common by EGF and IL-1 is sparser – fewer AP1 proteins were identified. We note, parenthetically, that the kinases, and even more the transcription factors activated solely by IFNγ or by IL-1 (i.e., those not in common with the EGF-activated set) are very distinct. This suggests that the effects of EGF overlap the distinct subset of the effects of IFNγ and of IL-1.

Certain genes induced by EGF are suppressed by EphrinAs, and *vice versa* (Figure 2). We reported that EphrinAs induce keratinocyte differentiation markers [38,44]; here we show that these markers are suppressed by EGF. The antiparallel effects are evident at both early and late time points (Table 4). SP600125 also induces keratinocyte differentiation [40]; correspondingly, similar antiparallel effects are seen in the EGF- and SP600125-treated keratinocytes (Table 4). Moreover, SP600125 induced inhibitors of proliferation, another effect antiparallel to EGF. Interestingly, the antiparallel effects of EphrinAs and SP600125 are distinct and seem to proceed *via* different mechanisms: of the kinases targets induced by EGF, EphrinAs seem to suppress the targets of MAPK-1, -3 and −14, while SP600125 none at all. Conversely the targets of CDK1 and CDK2 suppressed by EGF at late time point, seem induced by SP600125, but not by EphrinAs (Table 5). Again, the effects of EGF overlap the distinct effects of EphrinAs and of the JNK inhibitor SP600125.

**Table 5:** Kinases with common targets encoded by the EGF-, EphrinAs- and SP600125-regulated genes

**Kinase**	**EGF 1&4 h UP**	**SP 1&4 h DN**	**EFNAs DN**
MAPK1	2.51E-07	1	1.22E-02
SRC	5.21E-06	1	1
MAPK14	1.36E-05	1	1.88E-04
EGFR	1.39E-05	1	1
INSR	1.02E-04	1	1
MAPK3	1.70E-04	1	5.05E-02
JAK2	3.57E-04	1	1
ROCK1	2.62E-03	1	1
JAK1	3.30E-03	1	1
PTK2	3.62E-03	1	1
MAPK8	2.92E-02	1	1
PRKCQ	4.00E-02	1	1
ERBB2	5.35E-02	1	1
FYN	5.36E-02	1	1
JAK3	5.45E-02	1	1
IGF1R	7.63E-02	1	1
PRKCD	9.07E-02	1	1
	**EGF 24&48 h DN**	**SP 24&48 h UP**	**EFNAs UP**
CDK2	5.84E-03	1.43E-04	1
CDC2	1.21E-02	2.45E-06	1

UP denotes induced, DN suppressed genes. All kinases with p-values better than 0.1 for the EGF-regulated targets are listed. No kinases common to all three agents were identified.

An interesting paradox emerges from the comparison of genes regulated by EGF and by RA (Table 6). At the earliest time point, 1 h after addition of RA, the effects are largely antiparallel; for example, cell motility genes are inhibited by RA, while EGF induces those (Table 6a). At 4 h, both RA and EGF in parallel induce transcription factors and signal transduction and in parallel suppress apoptosis genes (Table 6b). Similar parallel effects are maintained after 24 h, when cell adhesion and cell motion seems induced by both EGF and RA. Interestingly, while MAPKs seem suppressed by RA after 1 h, they appear activated after 4 h. Their activation is not maintained at later time points, e.g., at 24 h, when targets of kinases EGFR, ErbB2 and Insulin receptor appear specifically induced in the EGF treated keratinocytes (Table 6c). The only transcription factors regulated by both EGF and RA to a statistically significant level are the NFκB proteins REL/RELA (Table 6d).

**Table 6:** Biological processes common in the EGF- and Retinoic acid-regulated genes

			
**a**			
**Gene ontology: Biological process**	**EGF1&4hUP**	**RA 1hDN**	
regulation of cell motion	1.45E-07	5.00E-06	
regulation of cell proliferation	1.65E-07	3.06E-04	
biological_process	5.84E-07	1.68E-03	
regulation of cell migration	6.33E-05	1.69E-05	
positive regulation of cell motility	6.15E-04	1.43E-02	
negative regulation of programed cell death	6.27E-04	4.43E-02	
positive regulation of cell migration	4.30E-03	7.98E-03	
anatomical structure development	5.71E-02	5.33E-03	
regulation of catalytic activity	7.77E-02	3.55E-02	
**b**			
**Gene ontology: Biological process**	**EGF1&4hUP**	**RA4hUP**	
regulation of signal transduction	1.41E-07	4.46E-04	
biological_process	5.84E-07	7.73E-05	
regulation of gene expression	2.27E-05	1.05E-04	
signal transduction	3.62E-05	3.42E-04	
cell communication	4.12E-05	2.77E-03	
response to stress	6.39E-05	8.06E-04	
regulation of transcription	7.87E-05	2.86E-04	
negative regulation of programed cell death	6.27E-04	4.51E-04	
regulation of apoptosis	8.12E-04	4.33E-02	
response to external stimulus	2.35E-03	4.24E-02	
negative regulation of apoptosis	3.07E-03	3.61E-04	
positive regulation of signal transduction	3.47E-03	4.51E-04	
positive regulation of metabolism	4.70E-03	1.99E-02	
regulation of RNA metabolic process	8.79E-03	4.78E-03	
phosphate metabolic process	1.08E-02	8.40E-05	
regulation of transcription	1.33E-02	6.50E-03		
regulation of transcription	1.59E-02	5.92E-03		
positive regulation of gene expression	3.33E-02	1.35E-02		
protein metabolic process	5.42E-02	1.39E-03		
positive regulation of transcription	9.37E-02	7.29E-03		
	**EGF1&4hUP**	**RA24hUP**		
biological_process	5.84E-07	1.20E-08		
regulation of gene expression	2.27E-05	1.57E-02		
signal transduction	3.62E-05	5.27E-04		
cell communication	4.12E-05	1.41E-03		
response to stress	6.39E-05	3.79E-04		
regulation of transcription	7.87E-05	6.83E-02		
negative regulation of programed cell death	6.27E-04	3.76E-04		
regulation of apoptosis	8.12E-04	1.03E-04		
response to wounding	8.17E-04	5.89E-04		
response to external stimulus	2.35E-03	4.81E-03		
regulation of cell adhesion	3.01E-03	3.76E-02		
negative regulation of apoptosis	3.07E-03	2.99E-04		
positive regulation of signal transduction	3.47E-03	3.66E-02		
cell motion	3.70E-03	7.55E-05		
positive regulation of metabolism	4.70E-03	7.30E-03		
phosphate metabolic process	1.08E-02	5.42E-02		
protein metabolic process	5.42E-02	6.91E-03		
	**EGF24&48hUP**	**RA24hUP**		
cell differentiation	5.90E-05	1.23E-02		
biological_process	1.32E-02	1.20E-08		
metabolic process	3.16E-02	1.43E-03		
**c**				
**Kinase**	**EGF1&4hUP**	**RA1hDN**		
MAPK1	2.51E-07	3.69E-03		
MAPK14	1.36E-05	3.33E-03		
MAPK3	1.70E-04	9.04E-05		
	**EGF1&4hUP**	**RA4hUP**		
MAPK1	2.51E-07	3.31E-05		
MAPK14	1.36E-05	1.45E-03		
EGFR	1.39E-05	6.66E-05		
MAPK3	1.70E-04	2.29E-05		
	**EGF1&4hUP**	**RA24hUP**		
EGFR	1.39E-05	2.17E-06		
INSR	1.02E-04	2.86E-05		
ERBB2	5.35E-02	8.18E-03		
FYN	5.36E-02	2.91E-02		
**d**				
**T. F.**	**EGF1&4hUP**	**RA4hUP**	**RA24hUP**	
RELA	2.28E-03	3.99E-02	3.34E-02	
REL	4.41E-03	4.19E-05	9.16E-02	

The early EGF-induced genes have extensive overlaps with both induced and suppressed genes by RA. a) Common processes in the EGF-induced and RA-suppressed genes 1 h after RA treatment. b) Common processes in the EGF-induced and RA-induced genes 4 h after RA treatment. c) Common kinase targets in the products of EGF-induced and RA-regulated genes. d) Only the NFkB family transcription factors have binding site overrepresented in both the EGF-induced and RA-regulated genes.

### Metaanalysis, part 2: EGF effects in keratinocytes *vs.* EGF effects in other cell types

EGF is a catholic regulator affecting many different cell types. Curiously, transcriptional responses to EGF have been described in several established cell lines, but not in primary cultures. We compared our data with EGF-regulated genes from 4 published studies, GSE6783, GSE6784, GSE10778 and GSE13009 [13,14,45]. These studies were selected because they compare directly EGF-treated *vs.* untreated cells and, for ease of comparison, because they used Affymetrix microarrays.

**Figure 4:**
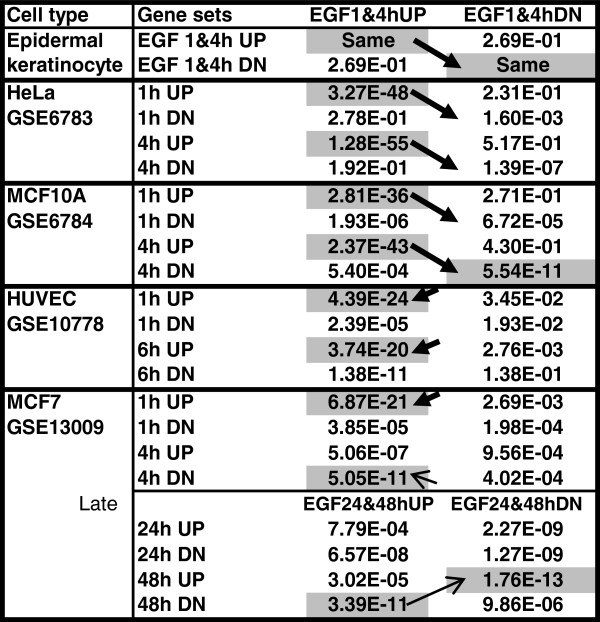
**Overlaps of the lists of genes regulated by EGF in keratinocytes with those regulated in other cell types.** The overlaps with p-values better than 10^-10^ are marked with gray. Downward arrows mark strong overlaps in both the induced and the suppressed gene lists. Upward arrows mark the antiparallel overlaps, i.e., genes induced in keratinocytes, but suppressed in MCF7 cells. The short arrows point to significant overlaps in the induced genes only, but not in the suppressed ones.

Interestingly, we find that in epidermal keratinocytes, as well as in HeLa and MCF10A cell lines, EGF regulates many genes in parallel at the early time points, 1 and 4 hrs (Figure 4). While in keratinocytes there is a very significant overlap between genes regulated at early and at late time points (Figure 4 top), the parallel with HeLa and MCF10A is clear for the induced genes only, not for the suppressed ones. Similarly, the parallel between keratinocytes and HUVEC holds for the induced genes only. In contrast, MCF7 cell line shows transcriptional effects antiparallel to those of keratinocytes, particularly at the later time points, at 48 h.

We find important parallels in the biological processes regulated by EGF in different cell types (Table 7). Specifically, regulation of transcription is affected in all cell types compared, at all time points (Table 7a). Similarly, negative regulators of apoptosis are induced in all cell types, although perhaps a little delayed in HUVEC (Table 7e). On the other hand, regulation of cell motility, signal transduction and cell proliferation are regulated in all four epithelial cell types, but not in the endothelial cells, HUVEC (Table 7b, c, d). Please also note that in MCF7, transformed cells, the regulators of transcription are downregulated at 48 hrs (marked with ! in Table 7).

**Table 7:** Biological processes common in keratinocytes and other cell types

	**Gene ontology process**	**Keratinocytes**	**HeLa**		**MCF10A**		**HUVEC**		**MCF7**		
		**EGF1&4hUP**	**GSE6783_1hUP**	**4 h UP**	**GSE6784_1hUP**	**4 h UP**	**GSE10778_1hUP**	**6 h UP**	**GSE13009_1hUP**	**4 h UP**	**48 h DN (!)**
a*	regulation of gene expression	2.27E-05	8.60E-07	5.40E-10	9.30E-07	7.98E-07	1.34E-07	1.42E-04	1.50E-12	2.22E-14	2.18E-04
	regulation of transcription	7.87E-05	9.91E-08	2.90E-10	1.48E-06	3.52E-07	1.42E-09	4.16E-06	3.00E-11	1.71E-13	5.94E-05
	regulation of RNA metabolic process	8.79E-03	1.64E-05	3.38E-08	1.44E-04	8.88E-05	1.83E-06	3.71E-04	1.47E-09	1.83E-11	3.06E-03
	regulation of transcription	1.33E-02	1.15E-04	2.32E-08	1.43E-04	9.91E-05	3.02E-07	8.11E-05	8.37E-09	1.32E-10	1.23E-03
	regulation of transcription	1.59E-02	2.54E-05	1.99E-09	7.78E-04	1.41E-02	4.72E-06	4.61E-06	4.42E-06	6.28E-05	2.24E-03
	positive regulation of gene expression	3.33E-02	9.78E-01	4.61E-03	1.24E-04	6.09E-05	2.04E-04	1.67E-01	2.38E-02	2.64E-04	1.19E-01
	positive regulation of transcription	9.37E-02	6.97E-01	2.09E-03	1.51E-04	5.38E-05	2.25E-04	8.84E-02	2.47E-02	1.73E-04	7.71E-02
**b**	regulation of signal transduction	1.41E-07	3.63E-03	1.89E-03	6.60E-05	2.12E-04	1	1	9.89E-07	1.93E-04	1
	signal transduction	3.62E-05	7.57E-02	6.05E-07	7.21E-11	4.10E-08	1	1	3.49E-10	4.23E-17	1
	positive regulation of signal trans.	3.47E-03	2.41E-01	3.57E-03	4.67E-03	6.83E-02	1	1	1.39E-01	6.02E-01	1
	response to external stimulus	2.35E-03	4.06E-01	1	8.03E-07	2.10E-01	1	1	1	1	1
	negative regulation of signaling	9.10E-05	1	1	1	8.38E-01	1	1	1	1	1
**c**	regulation of cell motion	1.45E-07	2.89E-02	4.73E-04	1.24E-02	5.93E-04	1	1	3.63E-04	6.91E-03	5.47E-01
	regulation of cell migration	6.33E-05	1.23E-02	6.66E-03	5.19E-02	1.44E-03	1	1	2.50E-04	3.01E-03	1
	positive regulation of cell motility	6.15E-04	2.41E-01	2.63E-02	6.38E-01	1	1	1	5.45E-03	7.82E-03	1
	positive regulation of chemotaxis	2.38E-04	9.30E-02	2.13E-03	1	1	1	1	5.00E-01	4.97E-02	1
	positive regulation of cell migration	4.30E-03	1.40E-01	9.60E-03	1	1	1	1	1.64E-03	1.73E-03	1
	cell motion	3.70E-03	1.13E-01	7.39E-02	4.32E-01	2.75E-01	1	1	1	6.20E-01	1
**d**	regulation of cell proliferation	1.65E-07	6.74E-03	1.76E-03	2.75E-03	6.40E-02	1	1	8.19E-04	1.59E-03	1
	positive regulation of cell proliferation	1.64E-03	4.97E-02	9.70E-04	8.01E-03	1.51E-01	1	1	8.50E-03	4.71E-01	1
**e**	negative regulation of prog. cell death	6.27E-04	1.86E-02	1.25E-03	1.58E-04	2.33E-04	1	4.82E-01	5.84E-02	2.87E-03	1
	regulation of apoptosis	8.12E-04	6.24E-02	8.43E-05	3.80E-07	2.73E-06	1	8.22E-02	4.64E-03	4.10E-05	1
	negative regulation of apoptos	3.07E-03	6.10E-02	2.80E-03	1.22E-04	4.83E-04	1	4.05E-01	4.69E-02	2.05E-03	1
	positive regulation of metabolism	4.70E-03	4.24E-02	5.71E-04	2.01E-05	6.52E-09	1.68E-03	1	4.33E-04	4.40E-07	3.26E-03
	regulation of catalytic activity	7.77E-02	6.24E-01	2.05E-04	2.31E-03	1.41E-06	1	1.41E-03	1.36E-04	2.64E-05	1
	cell communication	4.12E-05	7.24E-02	2.42E-06	3.87E-11	9.10E-07	1	1	2.48E-09	1.97E-15	1
	phosphate metabolic process	1.08E-02	4.54E-02	4.48E-06	1.12E-02	2.46E-04	1	1	5.72E-06	2.70E-06	1
	response to stress	6.39E-05	8.57E-02	5.22E-02	2.15E-07	7.26E-07	3.42E-02	1	1	1	1
	anatomical structure development	5.71E-02	1	2.87E-01	2.01E-02	1	1	1	1	5.53E-02	1
	transforming growth factor beta pathway	8.47E-02	1.19E-01	1	4.14E-01	1	4.44E-03	1	1	1	2.94E-01
	positive regulation of leukocycytes	1.40E-02	1	5.51E-01	2.75E-01	1	1	1	9.41E-02	1	1
	cell adhesion	8.83E-03	1	1	5.33E-04	1	1	1	1	1	1
	transmembrane receptor protein	1.49E-02	4.44E-01	1	8.14E-02	1	6.97E-02	1	1	1	1
	response to wounding	8.17E-04	1	1	2.40E-05	2.37E-01	1	1	1	1	1
	regulation of cell adhesion	3.01E-03	1	1	7.25E-05	6.46E-02	1	1	1	1	1
	negative regulation of DNA binding	9.91E-02	1	1	1	1	1	1	1	1	3.53E-01
	biological process	5.84E-07	1.83E-04	3.12E-14	3.61E-13	9.62E-15	6.34E-09	1.21E-07	1.43E-15	1.38E-21	3.52E-02
	protein metabolic process	5.42E-02	1	1.54E-06	4.66E-04	6.72E-06	1.27E-06	8.12E-02	2.46E-05	5.42E-08	6.91E-01
		**EGF 24&48 h UP**									**48 h DN (!)**
	biological process	1.32E-02	1.83E-04	3.12E-14	3.61E-13	9.62E-15	6.34E-09	1.21E-07	1.43E-15	1.38E-21	1.08E-06
	metabolic process	3.16E-02	1	4.33E-12	4.76E-05	2.73E-13	5.14E-15	6.13E-12	2.61E-05	2.83E-11	1.41E-07

The rightmost column shows the antiparallel overlap, i.e., with the genes suppressed by EGF in MCF7 cells. * Letters a – e indicate the fully or largely redundant biological processes.

## Discussion

To define comprehensively the molecular effects of EGF in human epidermal keratinocytes we used transcriptional profiling and identified the regulated genes. We compared these with genes regulated in keratinocytes by other extracellular signals, and with genes regulated by EGF in other cell types. We found both expected and unexpected classes of regulated genes. Because EGF regulates keratinocyte proliferation, the cell cycle genes are prominently regulated, as expected; conversely, the apoptotic signal genes are suppressed. Similarly, numerous genes related to motility and substrate attachment are induced by EGF, whereas those promoting rigid cell-to-cell contacts are suppressed. As expected from such a wide-ranging regulator, EGF induced many signalling proteins, both intra- and extracellular. Unexpectedly, EGF induced multiple members of the DUSP family; an important function of the DUSPs is to attenuate and dampen the signalling *via* ERKs, kinase members of the EGF-responsive signalling cascades. Thus, EGF induces its own feed-back mechanism, presumably to restrain uncontrolled proliferation, motility etc. At the same time, EGF induces production of HB-EGF and TGFα, ligands of EGFR, which are expected to augment and extend the signalling. A similar incongruity is seen at the late time points, 24 and 48 h, when both the cell cycle and the apoptosis genes are suppressed by EGF. Apparently, both positive and negative feedback are induced by EGF, and which will prevail may depend on additional, EGF-independent signals affecting the keratinocytes.

While keratinocytes in healthy epidermis divide in the basal layer and differentiate as they move outward to the surface of the skin, in wound healing and in various pathological conditions keratinocytes become ‘activated’ – they hyper-proliferate, migrate and elaborate complex defence mechanisms to protect the tissues and the organism beneath [46]. EGF is one such keratinocyte activating signal and induces production of proinflammatory and angiogenic cytokines and growth factors. Unexpectedly, EGF suppresses the production of antibacterial defensins and certain immunomodulators. Perhaps, then, the primary function of EGF in keratinocyte activation is in wound healing and re-epithelialization, rather than in immune responses and in fighting infection. We find that some of the activation-associated subsets of genes are co-regulated by EGF and IFNγ, some by EGF and IL-1 and yet other gene subsets by all there. This reflects the different basic functions of proliferative, inflammatory and immunomodulating cytokines and growth factors.

The regulation of keratinocyte differentiation markers by EGF is complex: while the expression of one subset of markers is suppressed, another subset is induced. Proliferation and differentiation are incompatible: promoting cell proliferation diverts the cells from differentiating and consequently, certain differentiation markers are suppressed by EGF. Unexpectedly however, lipid and steroid biosynthesis enzymes are induced by EGF. Lipids, including steroids, are essential for epidermal barrier function and are necessary for cornification, the final stage of epidermal differentiation. Lipid biosynthesis enzymes would not be expected to interfere with cell proliferation, unlike e.g., large keratins or filaggrin, and thus may not be incompatible with the proliferative effects of EGF in keratinocytes. While both EGF and RA generally inhibit keratinocyte differentiation, paradoxically, at the earliest time points many of the genes induced by one are suppressed by the other and *vice versa*. Only at later time points do EGF and RA regulate parallel sets of genes. Conversely, SP600125 and EphrinAs promote keratinocyte differentiation. However, the differentiation-associated genes affected by SP600125 and those affected by EphrinAs seem to be targets of different signalling pathways. These results suggest that epidermal differentiation is ‘modular’, not a fully integrated all-or-nothing process; for example, while subsets of differentiation markers are suppressed by EGF, other subsets are induced.

Importantly, metaanalysis of EGF regulation in keratinocytes and other cell types shows important parallel and antiparallel effects. Keratinocytes, HeLa and MCF10A cells show the highest parallelism. All three are epithelial and non-tumorigenic, and in these three cell types signal transduction, proliferation, motility etc. are regulated in parallel. In contrast, in HUVEC, which are not epithelial but endothelial cells, motility, proliferation and signal transduction are regulated differently. MCF7 cell line presents an interesting paradox: it is epithelial but also tumorigenic, migratory and invasive. While some of the early responses to EGF are parallel in MCF7 cells to those in other epithelial cell types, at late time points they become antiparallel – genes and processes induced by EGF in keratinocytes, HeLa and MCF10A are suppressed in MCF7, and *vice versa*. These conclusions agree with previous results demonstrating characteristic cytoskeleton proteins, attachment and cell spreading in the MCF7 cells [47,48].

## Conclusions

In conclusion, we present here a paradigm for systems biology approach for integration of disparate sources of transcriptional data. We used the effects of EGF in its eponymous tissue, defined the transcriptional profile changes and compared these with transcriptional profile changes in the same cell type by different signalling, and by the same signal in different cell type. The approach can be widely generalized for similar studies.

Specifically, we find that EGF induces characteristic aspects of keratinocyte activation, comprising proliferation and motility, but not activation of the immune response. These aspects partially overlap the effects of IFNγ and IL-1. At the same time, EGF suppresses certain aspects of keratinocyte differentiation, namely expression of many known differentiation markers, but not of others, e.g., lipid and steroid biosynthesis enzymes. We conclude that both activation and differentiation of keratinocytes are multifaceted, modular processes, rather than all-or-nothing events, and that distinct, characteristic and overlapping subsets of genes are regulated by different extracellular signals.

## Methods

### Human keratinocyte cultures

The growth and treatment of keratinocytes has been described [38,42,49]. Briefly, normal human neonatal foreskin epidermal keratinocytes were obtained from Dr. M. Simon (Living Skin Bank, Burn Unit, State University of New York, Stony Brook, NY) and grown in a defined serum-free keratinocyte growth medium (Keratinocyte-SFM, Invitrogen) supplemented with 2.5 ng/ml epidermal growth factor, 0.05 mg/ml bovine pituitary extract, and 1% penicillin/streptomycin (keratinocyte growth medium) at 37°C in 5% CO2 incubator. Generally, third passage cells were used at 70–80% confluence. We changed the medium to EGF-free and pituitary extract-free medium 24 h before the EGF treatment to starve the cells for EGF and to avoid the effects of the supplements in growth medium. Keratinocytes, grown on 100-mm plates, were then treated with human EGF (20 ng/ml, Invitrogen) or left untreated as controls. Parallel cultures of treated and control cells were harvested by scraping 1, 4, 24 and 48 h after treatment.

In our previous studies we used the same protocol for the effects of Interferon-γ, Interleukin-1, and Ephrin As, namely, the keratinocytes were pre-incubated in EGF-free, pituitary extract-free medium for 24 h before the addition of these agents [38,41,42]. In our experiments using SP600125 and retinoic acid we did not pre-incubate the cells but added the drug directly to the complete medium, i.e., containing both EGF and pituitary extract [39,40].

### Cell count and the migration assay

Keratinocytes were grown on 12-well plates to 80% confluency. The cells in un-supplemented medium were treated with EGF, or left untreated as controls. After 24 and 48 h the cells were trypsinized and counted using a hemocytometer. Three wells were measured at each time point in each treatment. The error bars represent the highest and lowest measured values.

Keratinocytes were grown on 12-well plates up to 80-90% confluency as above, and treated with 8 μg/ml mitomycin-C for 1 hour to prevent cells from dividing. After washing, the keratinocyte monolayers were scratched with a 200 μl pipette tip in each well to form a cross. Then EGF (20 ng/ml) was added with the fresh medium. Media were changed every 24 hours. At the 0, 24, and 48 h time points after the treatment, pictures were taken under a microscope.

### Preparation of labelled cRNA and geneChip hybridization

We isolated total RNA from treated and untreated keratinocytes using RNeasy kits (Qiagen) according to the manufacturer’s instructions. Approximately 5 μg of total RNA was reverse transcribed, amplified, and labelled as described [49]. Fifteen micrograms of labelled cRNA was fragmented and hybridized to HG-U95Av2 arrays (Affymetrix). Arrays were washed, stained with anti-biotin streptavidin-phycoerythrin-labelled antibody, and scanned using the Agilent GeneArray scanner system (Hewlett-Packard). A single array was hybridized for each of the 8 culture conditions.

### Array data analysis

**Figure 5:**
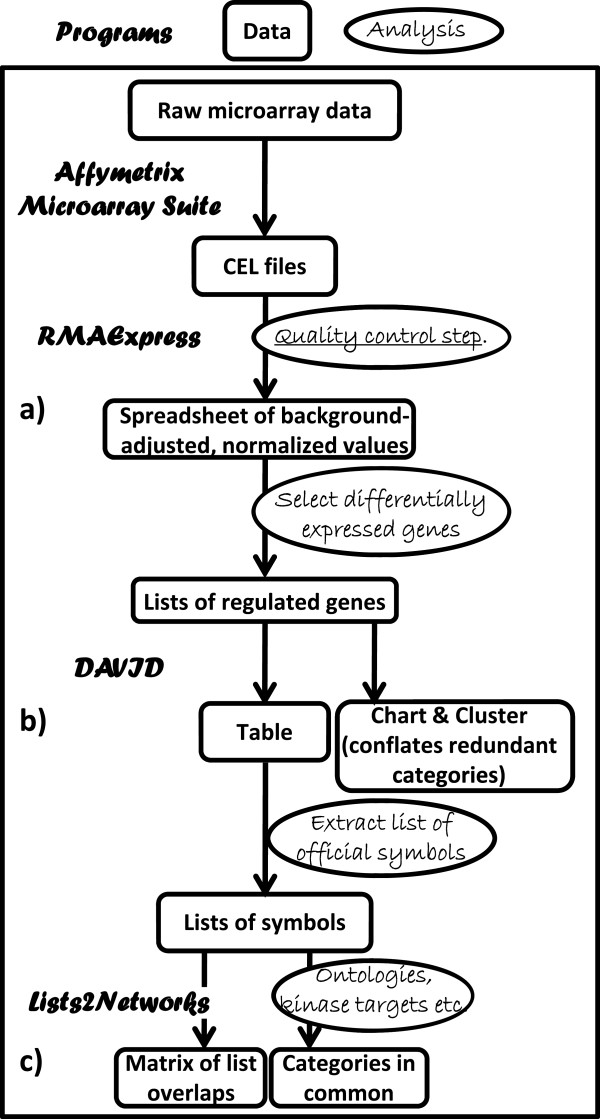
**Flowchart of analysis.** The data flow from rectangle to rectangle; the programs are named in bold italics and the analyses to be performed by the investigator using spreadsheet are in ovals. **a)** An example of large spreadsheets is given in Additional file 1: Table S1. **b)** An example of Tables is given in Additional file 2: Table S3, of Charts in Table 1 and of Clusters in Additional file 5: Table S2**. c)** An example of Matrices is given in Figure 2, of Kinase Targets in Table 5 and of Ontological Categories in common in Table 7.

The flowchart of our analysis is presented in Figure 5. Intensity values from the chips were obtained using Microarray Suite version 5.0 (Affymetrix) and scaled by calculating the overall signal for each array. Raw data CEL files have been deposited in the Gene Expression Omnibus (GEO) database (pending). To compare data from multiple arrays, the signal of each probe array was scaled to the same target intensity value. RMAExpress [50] was used for background adjustment, quintile normalization, summarization and quality analysis. Regulated genes were selected as differentially expressed 50% or more between the EGF-treated and the control samples at the same time point. Annotation and ontology of the regulated genes was obtained using the Database for Annotation, Visualization and Integrated Discovery, DAVID [24]. DAVID provided ‘tables’ containing functional and ontological details of the regulated genes, ‘charts’ containing ontological categories, pathways etc., over-represented in the gene lists, ‘clusters’ of such ontological categories (which identified redundancies and overlaps), transcription factors over-represented in the promoters of the genes, as well as sub-lists of genes specific for each ontological category. Transcription factor binding sites were also evaluated using DAVID, in a separate analysis.

### Comparisons of regulated genes in keratinocytes

In our previous studies we defined the genes regulated in keratinocytes by Interferon-γ, Interleukin-1, retinoic acid, EphrinA proteins and JNK inhibitor SP600125 [38-42]. Using DAVID, we collected the lists of official symbols of regulated genes in those studies, as well as of the EGF-regulated genes from this study and submitted them to the Lists2Networks analysis program [43]. The program compares lists for mutual overlaps within specific categories, e.g., targets of protein kinases, ontological biological process or OMIM disease gene association, returning statistical evaluation of the overlaps. While we analyzed 7 different relevant categories, we find ‘Gene Ontology Biological Process’ to be the most informative, probably because it is the best-annotated and most complete. We downloaded the matrices of p-values of gene lists overlaps, as well as spreadsheets of p-values of individual biological processes, Bonferoni-corrected for multiple comparisons.

### Metaanalysis of transcriptional responses to EGF in various cell types

We searched GEO datasets and ArrayExpress using “EGF” and “human” as keywords; then manually selected the studies that directly compared EGF-treated *vs.* nontreated samples. For simplicity of comparison, we limited ourselves to the studies using Affymetrix microarrays. For four such studies we downloaded the CEL files and analyzed those using RMAExpress [50]. We then eliminated the outlier chips, i.e., those with Normalized Unscaled Standard Error medians 5% or more different from other chips. This procedure provides a measure of relative chip quality derived from the residuals from the RMA model (for details see ref. [50]). After identifying the outliers, we have re-run the RMAExpress program without the outliers. The EGF-regulated genes were selected as described above. Lists of Affymetrix IDs of regulated genes were submitted to DAVID [24]. From the tables provided from DAVID analysis, we extracted lists of consensus gene symbols, which we submitted to the Lists2Networks program [43]. This allowed us to identify specific functional commonalities in various lists of genes, providing the metaanalysis results. Additional details of dataset search, selection procedures and criteria, quality control, data formats, ontological analyses and metaanalyses will be published elsewhere (M.B. and C. Mimoso, in preparation).

## Competing interests

The author declares that he has no competing interests.

## Author’s contribution

M.B. conceived the study, analyzed the results and wrote the paper.

## Supplementary Material

Additional file 1: Table S1 EGF regulated genes. Expression levels are given in Log_2_-transformed values. C denotes control, untreated, E the EGF-treated samples. Red colour identifies genes induced by EGF 2-fold or more, pink those induced 50% to 2-fold; bright green mark genes suppressed 2-fold or more, light green those suppressed 50% to 2-fold. We also show the maximum level of expression, and the maximum, minimum and average levels of regulation. In the second spreadsheet of the Additional file 1: Table S1 only the genes regulated at least 50% are listed, the unselected genes are not. Click here for file

Additional file 2: Table S3 EGF regulated genes in specific ontological categories regulated by EGF at early time points, 1 and 4 h. a-f EGF induced genes. g-k EGF suppressed genes. For details, please see the main text. Note that many genes belong to multiple categories. Click here for file

Additional file 3: Table S4 EGF regulated genes in ontological categories regulated by EGF at late time points, 24 and 48 h. a-f EGF-induced genes. g-i EGF-suppressed genes. Click here for file

Additional file 4: Table S5 Numbers of genes in analyzed lists. These lists were submitted to the Lists2Networks program. Results of analyses are given in Figures 2 and 4, Tables 5 and 7. Click here for file

Additional file 5: Table S2 Clustering of categories of EGF regulated genes. DAVID algorithm identifies ontological and other categories that have statistically significant overlaps of gene lists; this usually folds redundant categories into easy-to-interpret clusters. Only clusters with highest enrichment scores are presented, and in each cluster only the top 2–3 categories. Count columns give the number of genes in each category, % the percentage of submitted genes that belong to that category, p-values the statistical value of each category (see e.g., Table 2 in the main text), while the enrichment score of a cluster gives the statistical significance of the overlaps of gene lists Click here for file
